# A shear gradient-activated microfluidic device for automated monitoring of whole blood haemostasis and platelet function

**DOI:** 10.1038/ncomms10176

**Published:** 2016-01-06

**Authors:** Abhishek Jain, Amanda Graveline, Anna Waterhouse, Andyna Vernet, Robert Flaumenhaft, Donald E. Ingber

**Affiliations:** 1 Wyss Institute for Biologically Inspired Engineering, Harvard University, Boston, Massachusetts 02115, USA; 2 Division of Hemostasis and Thrombosis, Department of Medicine, Beth Israel Deaconess Medical Center and Harvard Medical School, Boston, Massachusetts 02115, USA; 3 Vascular Biology Program, Departments of Pathology and Surgery, Boston Children's Hospital and Harvard Medical School, Boston, Massachusetts 02115, USA; 4 Harvard John A. Paulson School of Engineering and Applied Sciences, Harvard University, Cambridge, Massachusetts 02138, USA

## Abstract

Accurate assessment of blood haemostasis is essential for the management of patients who use extracorporeal devices, receive anticoagulation therapy or experience coagulopathies. However, current monitoring devices do not measure effects of haemodynamic forces that contribute significantly to platelet function and thrombus formation. Here we describe a microfluidic device that mimics a network of stenosed arteriolar vessels, permitting evaluation of blood clotting within small sample volumes under pathophysiological flow. By applying a clotting time analysis based on a phenomenological mathematical model of thrombus formation, coagulation and platelet function can be accurately measured *in vitro* in patient blood samples. When the device is integrated into an extracorporeal circuit in pig endotoxemia or heparin therapy models, it produces real-time readouts of alterations in coagulation *ex vivo* that are more reliable than standard clotting assays. Thus, this disposable device may be useful for personalized diagnostics and for real-time surveillance of antithrombotic therapy in clinic.

## Abstract (web summary)

The current hemostasis assays are unable to predict thrombotic or bleeding risk in clinics. Here, Jain *et al*. present a novel microfluidic device mimicking stenosed arterioles that determines clotting times *in vitro* and in extracorporeal circuits, offering a simple and reliable monitoring of blood homeostasis and platelet function.

## Graphical abstract

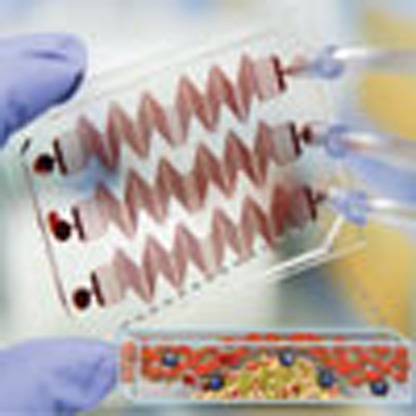

Rapid, quantitative and accurate haemostasis monitoring is critical in many clinical settings (for example, surgery, trauma, sepsis, anticoagulation and anti-platelet therapies) to anticipate, avoid and direct the management of serious disorders due to bleeding or thrombosis^1^,^2^,^3^. An increasing number of patients worldwide who are treated with the help of extracorporeal assist devices (for example, haemodialysis, membrane oxygenation, mechanical circulatory support, and so on) require precise and personalized anticoagulation dose monitoring on as close to real-time basis as possible to maintain haemostasis *in vivo*, and to prevent clotting in the blood transport lines and pumps of these devices *ex vivo*^4^,^5^. Most concerning is that the inability to manage anticoagulation dosage precisely and continuously is a common cause of catastrophic haemorrhage and mortality in patients^6^. Moreover, the requirement for monitoring haemostasis reliably at the bedside using low blood volumes is even more critical in paediatric patients with acute coagulopathy (acquired or congenital), as they are more vulnerable than adults^7^,^8^,^9^.

Various tests and devices have been developed over many decades to assess blood clotting and platelet function *in vitro*, including assays for bleeding time, activated clotting time (ACT), activated partial thromboplastin time (aPTT), thromboelastography and platelet aggregometry. While these tests provide useful information regarding coagulation status or platelet function, they are limited in terms of their ability to predict thrombotic or bleeding risk in clinical settings^10^,^11^,^12^ at least in part because they fail to incorporate many of the key contributors to haemostasis control that exist *in vivo*. For example, a major limitation of these assays is that they measure clotting behaviour under static (no flow) or irrelevant flow conditions, and thus, they fail to incorporate the effects of haemodynamic forces (pressure, flow and shear stress) and related cellular interactions that are known to significantly impact whole blood thrombosis in the living vasculature^3^,^13^. Notably, flow acceleration and deceleration (fluid shear gradients) have been shown to initiate platelet aggregation during arterial thrombosis *in vivo*^14^,^15^, and clotting in extracorporeal devices usually occurs at sites of sudden flow disturbances, stagnation points and stenosed sections of tubing^16^,^17^. However, none of the routinely used haemostasis assays incorporate these physiological conditions in their assessment of blood coagulation.

Microfluidic devices and parallel plate flow chambers that mimic atherosclerotic confinement of vessels and reconstitute physiological shear rates and gradients have been developed for basic research studies using human whole blood in combination with extracellular matrix (for example, collagen) surface coatings^15^,^18^,^19^. These studies confirmed that haemostasis induced by blood contact with matrix varies depending on local flow conditions; however, the basic insights gained from these studies have not been translated into clinical practice, mainly because these devices are not designed for bedside use or they require highly specialized instrumentation and imaging systems^19^,^20^,^21^,^22^,^23^. Here we describe a simple microfluidic device for real-time monitoring of haemostasis and platelet function that permits rapid determination of a clotting time for native whole blood as it is perfused through a network of channels, and mimics local physiologically relevant changes in haemodynamic flow conditions experienced in living stenosed arterioles. A major design advancement in this microfluidic device is that these channels include multiple pre-stenosed and post-stenosed regions in the flow stream that generate both pathophysiological shear rates and shear gradients, which act as haemodynamic activators to cause blood clotting inside the device. To accurately assess clot formation in an automated manner, changes in fluid pressure caused by increasing channel occlusion are tracked in real time in the device, and a physiologically relevant ‘microfluidic' clotting time is derived as a quantitative end point using a novel mathematical model. We demonstrate the sensitivity of this assay, and the reliability of this microfluidic clotting time *in vitro* using clinically relevant and patient samples. We also show that the device can be integrated directly into vascular access lines and blood-contacting medical devices for real-time *ex vivo* monitoring of changes in haemostasis within native flowing blood.

## Results

### Haemostasis monitoring microdevice

**Figure 1:**
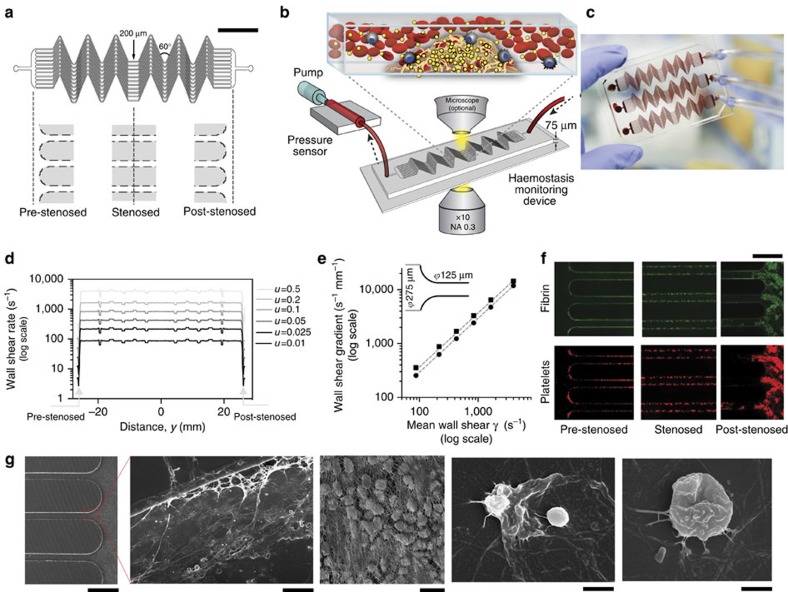
The biomimetic haemostasis monitoring microdevice. (**a**) Diagram of the device with line drawings below showing the design of the accelerating (pre-stenosed), uniform width (stenosed) and decelerating (post-stenosed) regions of the microchannels. The central stenosed region contains 12 parallel lanes of 200-μm-wide and 75-μm-high channels that repeatedly turn 60° a few times in each channel (scale bar, 500 μm). (**b**) Schematic of the haemostasis monitor device and method. Human whole blood is pushed or pulled by a syringe through an inline pressure sensor that connects to the device via tubing, and used to determine micro-clotting time. Optionally, fluorescence microscopy of fibrinogen and platelets allows simultaneous monitoring of thrombus formation. (**c**) Photograph of three haemostasis monitoring devices formed in a single piece of PDMS mould on top of a standard glass slide (75 × 50 mm). (**d**) Graph showing results of computational modelling of blood flow through the device. The wall shear rate across the length of the entire device (pre-stenosed through stenosed and post-stenosed; from left to right) is shown for various inlet flow velocities (*u*=0.01–0.5 m s^−1^). (**e**) The computed wall shear gradients at the pre-stenosed (black circles) and post-stenosed (black squares) sections plotted against the mean wall shear at the stenosed section (line of linear regression (dotted line); goodness of fit, *r*^2^=0.99; average slope, 3.49 mm^−1^). This linear relationship corresponds to a blood vessel that comprises of ∼55% stenosis. (**f**) Representative fluorescent micrographs of pre-stenosed, stenosed, and post-stenosed regions of the same microfluidic device, showing fibrin (top, green) and adhered platelets (bottom, red) after perfusing blood through the device, containing heparin (0.25 IU ml^−1^), for 20 min (scale bar, 500 μm). (**g**) Scanning electron micrographs of a blood clot formed inside the device near the post-stenosed region shown at progressively higher magnifications (left to right; scale bar, 500, 50, 10, 5 and 1 μm) demonstrating the presence of fibrin networks containing trapped blood cells (3 images at left), including activated platelets (2 right images).

We designed a microfluidic device containing microchannels that mimic stenosed arterioles (for example, narrowed due to atherosclerotic plaque formation) to create sudden fluid acceleration (pre-stenosis), followed by a region of uniform shear (stenosed region), and then by a region with a sudden deceleration (post-stenosis), when whole blood is perfused through the device (Fig. 1a). This was achieved by allowing the blood to first enter into a large reservoir (∼8 mm wide, 75 μm high) and then splitting the flow into 12 smaller parallel channels (200 μm wide, 75 μm high); followed by convergence of the flow into an outlet similar to the inlet (Fig. 1a). The 12-channel design was chosen to mimic a vascular bed containing a network of multiple small vessels, while simultaneously maximizing the surface area exposed to flowing blood to increase the likelihood of clot formation. This design also produced a high dynamic measurement range (∼0.4–12 p.s.i.) and a good signal-to-noise ratio when a commercially available pressure sensor was attached externally to the device (Supplementary Fig. 1). In addition, the total width and length of the device were designed to fit on a standard glass microscope slide to enable simultaneous real-time optical microscopic imaging using a low magnification objective, while ensuring near homogenous flow in all parallel channels (Fig. 1a,b). Each channel contained a few sections with alternating 60° bends and straight sections to achieve the highest possible surface contact area available to promote clot formation, and three replica devices were placed on each glass slide to permit three parallel measurements (replicates) at the same time (Fig. 1c). Finite element computational analysis of non-Newtonian blood flowing through the device confirmed that for a given flow velocity inlet boundary condition, the wall shear rate rapidly changes at the pre-stenosed and post-stenosed junctions over a distance of ∼300 μm, and remains mostly uniform in the straight section (Fig. 1d, Supplementary Fig. 2). These computational experiments also showed that a linear relationship exists between the wall shear rate (*γ*) at the straight section (determined by the applied flow rate) and the maximum shear rate gradient (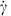) for this device design, due to flow acceleration and deceleration 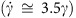 (Fig. 1e). Based on this finding, we calculated the difference in shear across the stenosed region in our device, and determined that it corresponds to a shear change equivalent to that produced when the diameter of an arteriole decreases from ∼ 275 to 125 μm (*ϕ*) or ∼55% stenosis, which is typically seen clinically, for example, in patients with arteriosclerosis^8^,^24^. In practice, we operated the device so that the flow rates were maintained within the range of 5–150 μl min^−1^, leading to wall shear gradients from ∼250 to 9,000 s^−1^ mm^−1^, which correspond to maximum wall shear rates from 75 to 2,500 s^−1^ (3–100 dynes cm^−2^) in the uniform region, which again is typical for an arteriolar bed. Flow rates outside this range also were undesirable as they would result in frequent red blood cell sedimentation and require larger volumes of human donor blood. In our experiments, when we added a low dose of heparin anticoagulant (0.25 IU ml^−1^) to native whole blood to prevent immediate clotting before infusion into the device, and after perfusing the blood through microfluidic device for ∼20 min, we detected formation of various sized clots throughout its entire length using fluorescence imaging. However, much more prominent and larger clots formed at the post-stenosed region where the flow suddenly decelerates (Fig. 1f) and we could visualize trapped blood cells, fibrin and elongated platelets within the growing thrombus using electron microscopy (Fig. 1g), confirming that near-physiological coagulation and platelet activation occur inside the device. Importantly, this finding is reminiscent of thrombus formation observed at stenosis sites *in vivo*, as well as inside extracorporeal circuits in the clinic.

### A mathematical formulation of clotting dynamics

**Figure 2:**
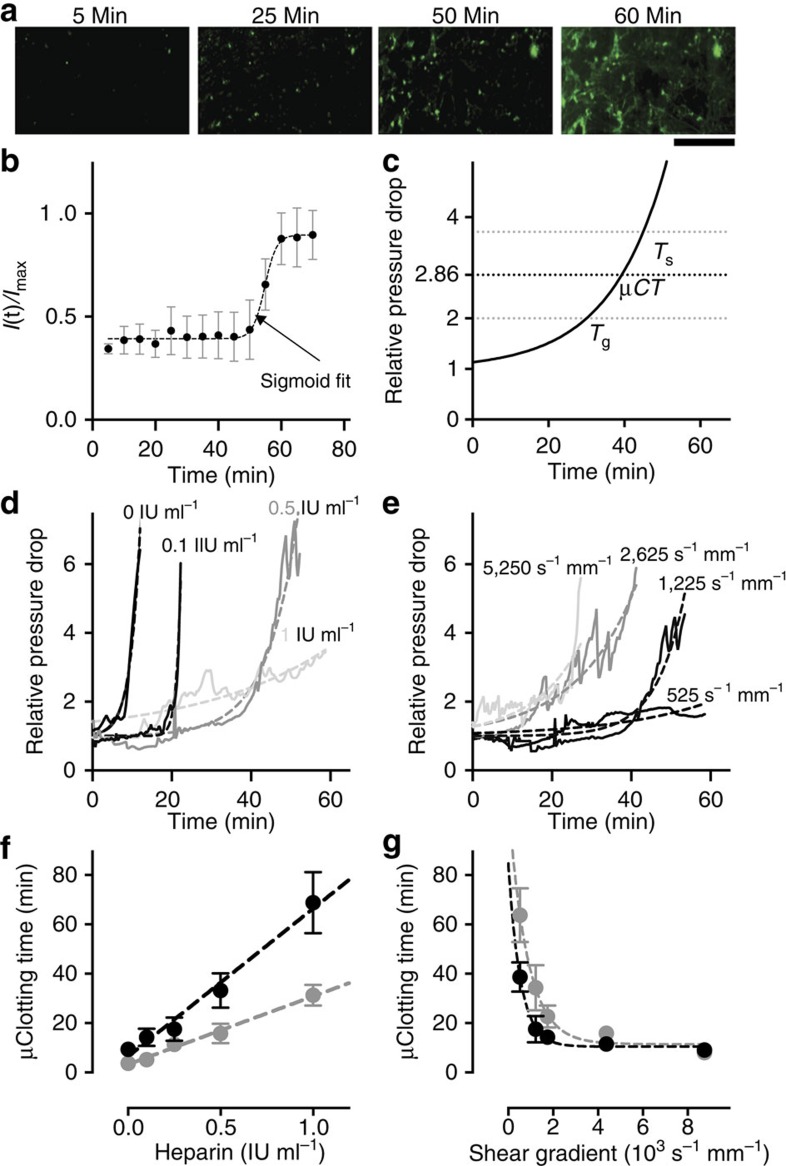
Mathematical formulation and empirical validation of clotting time. (**a**) Representative fluorescent micrographs of fibrin (green) deposited within the microchannel after blood containing heparin (0.75 IU ml^−1^) was perfused through the channel for 0, 25, 50 and 60 min at a shear rate gradient of 4,375 s^−1^ mm^−1^ (scale bar, 250 μm). (**b**) The fluorescence intensity of fibrin clot normalized by the maximum (black circles), showing a sigmoidal trend (dotted line, goodness of fit, *R*^2^=0.99; *n*=3 donors, 1 replicate per experiment) over time in the observed region. Due to sigmoidal thrombus growth, the pressure rise across the device (relative to the pressure at the initial time) will follow a two-parameter (*T*_g_ and *T*_s_) exponential (reciprocal of sigmoid) trend. (**c**) A representative plot showing mathematical function to predict thrombus formation with *T*_g_=30 min and *T*_s_=45 min. Microfluidic clotting time (μCT) is average of *T*_g_ and *T*_s_. (**d**,**e**) Representative graph showing empirical validation of the analytical model by varying heparin (**d**; 0, 0.1, 0.5 and 1 IU ml^−1^; labelled in different shades of grey) at a given shear rate gradient of 1,225 s^−1^ mm^−1^, and by varying shear rate gradient (**e**; 525;1,225;2,625 and 5,250 s^−1^ mm^−1^; labelled in different shades of grey) at heparin concentration of 0.5 IU ml^−1^. (goodness of fits for **d** and **e** are presented in Supplementary Table 1; three experiments were performed for every condition). (**f**,**g**) Graph showing empirical validation of μCT derived from the phenomenological analytical model, by varying the heparin concentration in the range of 0–1 IU ml^−1^ and perfusing blood at shear rate gradients of 1,225 s^−1^ mm^−1^ (black circles) and 4,375 s^−1^ mm^−1^, (grey circles) respectively (**f**; line of linear regression (dotted line); goodness of fit, *R*^2^=0.99 and 0.98, respectively; *n*=3 donors, 1 replicate per experiment); and by varying the shear gradient in the range 262–8,750 s^−1^ mm^−1^ and perfusing blood at heparin concentrations of 0.25 IU ml^−1^ (black circles) and 0.5 IU ml^−1^ (grey circles), respectively (**g**; exponential decay regression curve (dotted line); goodness of fit, *R*^2^=0.76 and 0.79, respectively; *n*=3 donors, 1 replicate per experiment).

The dynamics of fibrin clot formation in an arterial vessel *in vivo*, or in an *in vitro* hollow channel, consist of three stages—a steady reaction time, a growth phase and saturation (full stenosis)^25^. To explore the dynamics of clot formation in our microfluidic device, we performed time-lapse microscopic analysis of whole human blood containing both a typical therapeutic heparin dose (0.75 IU ml^−1^; ref. 26) and fluorescently labelled fibrinogen as it flowed through the channel and entered a post-stenosed region, at a pathologically relevant rate corresponding to a wall shear gradient of 4,375 s^−1^ mm^−1^ (Fig. 2a). When the mean fluorescence intensity, *I*(t), normalized by the intensity of a fully clotted region (*I*_max_), was plotted against time, we found that clotting followed a sigmoidal trend (Fig. 2a,b and Supplementary Movie 1) involving sequential phases of steady reaction, growth and saturation similar to those previously described^25^.

Based on this observation, we then used these sigmoidal dynamics to derive a mathematical formulation of clot formation (see Methods for details). The model led to the following relation to predict pressure rise in a microchannel undergoing occlusion when constant flow is applied (Fig. 2c):


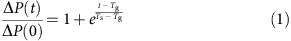


*T*_g_ and *T*_s_ are the characteristic parameters of the fitted two-parameter exponential growth curve (reciprocal of sigmoid) that represent time for the pressure to double and (1+e) times its initial value, respectively. For practical purposes, we derived a clotting time that is the average of *T*_g_ and *T*_s_, which we determined to be equal to the time when the pressure rises to 2.86 times the initial pressure (Fig. 2c).

We then experimentally tested the model by investigating clotting dynamics in flowing native human whole blood supplemented with different heparin concentrations when the flow rate was set to produce a shear gradient of 1,225 s^−1^ mm^−1^. The regression curve fits based on the mathematical model predicted the experimental data with a high accuracy (Fig. 2d, goodness of fits in Supplementary Table 1). To further determine the robustness of the model, we varied the fluid shear rate gradients at a constant heparin concentration (0.5 IU ml^−1^) and analysed the pressure rise measured with the inline pressure sensors; again, the regression curves accurately fit the experimental data (Fig. 2e, goodness of fits in Supplementary Table 1). Importantly, in all of the clotting time analyses done in this study over the entire range of heparin concentrations and shear gradients that we applied (Supplementary Table 1), we found that the goodness of fit (*R*^2^) was close to unity (0.89+0.11 s.e.m.), where *R*^2^=0 indicates the model is theoretically fully inaccurate and *R*^2^=1 represents a theoretically perfect model. Therefore, the microfluidic clotting time derived from these curves can act as a highly reliable quantitative end point for this clotting assay, including under conditions in which blood contains therapeutically relevant heparin concentrations (0–1 IU ml^−1^), and under variable shear conditions that occur within stenosed arterioles *in vivo* and that are the major sites of thrombosis in various types of extracorporeal devices.

### Microfluidic clotting time analysis

We next evaluated the reliability of the microfluidic clotting times derived from these regression models to determine if they can serve as quantitative end points in lab and clinical settings. First, we measured heparin sensitivity in the relevant concentration range (0–1 IU ml^−1^), and found that the clotting times accurately followed a linear increase as the heparin concentration was raised from 0 to 1 IU ml^−1^ at both low (1,225 s^−1^ mm^−1^) and high (4,375 s^−1^ mm^−1^) fluid shear gradient levels (Fig. 2f). This finding confirms that this device can be used to reliably monitor heparin therapy in its clinical range within blood samples *in vitro*.

Because current haemostasis tests used in the clinic do not incorporate the physiological contributions of haemodynamic shear stresses and gradients^27^, we explored whether we can detect changes in clotting times in response to pathophysiological variations in shear rates and gradients in our microdevice. Arterial clotting is dominated by platelet activation and aggregation that increase with shear^27^,^28^. Here, we observed an exponential decrease in microfluidic clotting time as the shear rate gradient increased from 525 to 8,750 s^−1^ mm^−1^ at heparin concentrations of 0.25 and 0.5 IU ml^−1^ (Fig. 2g), confirming that this microfluidic clotting time analysis is also sensitive to changes in platelet function due to variable fluid shear.

### A rapid *in vitro* platelet function test under stenosed flow

**Figure 3:**
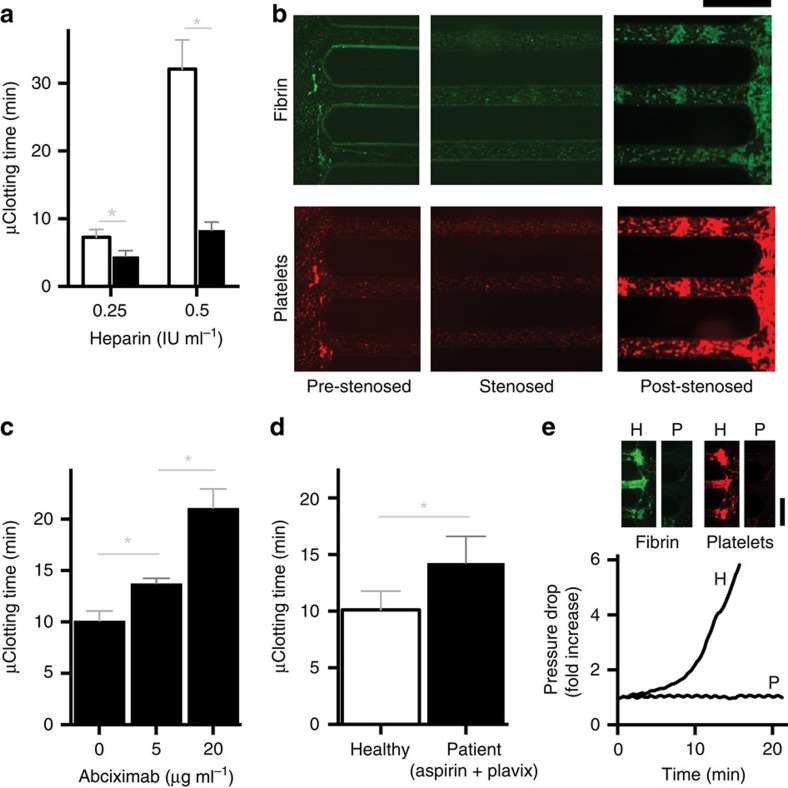
Rapid platelet function analysis *in vitro* (**a**) Microfluidic clotting time (μCT) measured when the blood is perfused (shear rate gradient=1,225 s^−1^ mm^−1^) through a haemostasis monitoring microdevice coated with collagen (black bars, 100 μg ml^−1^) compared with bare surface (white bars), shown at two different heparin concentrations (0.25 IU ml^−1^ and 0.5 IU ml^−1^, respectively; *P<0.05, multiple *t*-tests, s.e.m.; *n*=3 donors, 2 replicates per experiment). (**b**) Representative fluorescent micrographs of pre-stenosed, stenosed and post-stenosed regions of the same collagen-coated microfluidic device (shear gradient, 4,375 s^−1^ mm^−1^), showing fibrin (top, green) and adhered platelets (bottom, red) after it occluded due to perfusion of recalcified citrated blood for 20 min (scale bar, 500 μm). (**c**) μCT measured for citrated blood containing different doses of an anti-platelet drug, abciximab (Reopro), when perfused through the device after recalcification (shear gradient, 4,375 s^−1^ mm^−1^; *P<0.05, one-way ANOVA, s.e.m.; *n*=3 donors, 2 replicates per experiment). (**d**) μCT measured from perfused recalcified citrated blood samples (shear gradient, 4,375 s^−1^ mm^−1^) of healthy donors (white bars) versus patients who take Aspirin and Plavix (clopidogrel) regularly (black bars) (*P<0.05, unpaired *t*-test, s.e.m.; *n*=5 healthy donors and 9 patients, 2 replicates per experiment). (**e**) A representative trace of pressure rise measured in the device, and fluorescent micrograph showing fibrin (top left,green) and platelet (top right,red) deposition at the post-stenosed region of the device, when blood from a healthy individual (H) was flowed through the device compared with blood from an HPS patient (P; shear gradient: 4,375 s^−1^ mm^−1^; scale bar: 500 μm). HPS, Hermansky–Pudlak Syndrome.

As platelet aggregation is a major contributor to the development of vascular occlusion,^29^ we further explored if this biomimetic device can be used as a rapid platelet function assay under stenosed flow. First, to reduce the blood volume and time required for analysis, we functionalized the surface of the microchannel within the device with collagen, which is a commonly applied platelet agonist.^20^ This reduced the microfluidic clotting time significantly (*P*<0.05; multiple *t*-tests), thereby enabling us to use the device to perform clotting analysis in a few minutes using <1 ml of human whole blood (Fig. 3a). Notably, even after collagen coating, we still observed larger amounts of fibrin and platelet aggregates in the post-stenosed regions, which is consistent with past findings suggesting that the shear rate gradient in the decelerating flow region may be a major contributor to the haemostatic response^14^,^15^ (Fig. 3b). Next, we used the collagen-coated devices to analyse blood samples that were anticoagulated with sodium citrate, which were then reconstituted with calcium and magnesium immediately before infusion into the device to reactivate the coagulation cascade. When the anti-platelet drug abciximab (Reopro) that targets integrin α_IIb_β_3_ was added at clinically relevant increasing doses (0, 5 and 20 μg ml^−1^) to blood samples drawn from healthy individuals, we observed a dose-dependent rise in microfluidic clotting time (*P*<0.05; one-way analysis of variance (ANOVA); Fig. 3c). When we perfused blood from patients who were treated chronically with the dual anti-platelet regimen of aspirin (cyclooxygenase inhibitor) and clopidogrel (Plavix; P2Y_12_ inhibitor), we also detected a significant increase in clotting time relative to healthy individuals (*P*<0.05; unpaired *t*-test; Fig. 3d).

We then explored whether we could use this collagen-coated device to measure clotting defects in blood samples from patients with Hermansky–Pudlak syndrome (HPS), which is an extremely rare bleeding disorder characterized by a deficiency of platelet dense granules. Importantly, conventional standard platelet aggregation assays (PFA-100, bleeding time etc.) commonly do not detect the platelet defect in HPS patients, and neither these methods nor platelet aggregometry are recommended as a first line clinical test for this syndrome; as a result, time consuming and specialized electron microscopy is still required for an unequivocal diagnosis at this time^30^. Interestingly, when we perfused the device with blood from two HPS patients, we did not observe any clotting for the entire duration of the experiment in either subject, and their blood did not clot even when we applied a wall shear rate gradient as high as 8,750 s^−1^ mm^−1^ (Fig. 3e and Table 1). These findings show that the shear gradient-activated microfluidics device is highly sensitive in detecting platelet defects in these HPS subjects, and that the clinical manifestation of this disease (that is, failure to form clots normally within flowing blood *in vivo*) can be effectively recapitulated *in vitro* using this new method. This observation also inspired us to explore if occlusion in this device is dependent on the release of ADP agonist^31^,^32^ from platelet dense granules, which are dysfunctional in HPS patients. To test this, we added ADP to the blood of HPS patients and found that adding ADP reversed their clotting defect in the microfluidic device (Table 1), further confirming sensitivity to defective ADP release and providing another independent biomarker to evaluate bleeding in HPS patients.

Together, these proof-of-concept studies demonstrate that clotting time analysis in the microfluidic device is sensitive to platelet adhesion to collagen and modulation of platelet activation pathways that are most commonly targeted by current anti-platelet regiments, including the ADP-P2Y_12_ pathway, the cyclooxygenase pathway, and activation through α_IIb_β_3_. By studying a small number of HPS patients, we also demonstrated that this device can be used to detect this extremely rare condition, which cannot be easily detected by conventional platelet aggregation or blood clotting assays. Thus, this microfluidic haemostasis monitoring device potentially offers a convenient new method for global assessment of platelet function, monitoring anti-platelet therapies, and evaluating human patients with bleeding disorders.

### *Ex vivo* monitoring of coagulopathy and heparin therapy

**Figure 4:**
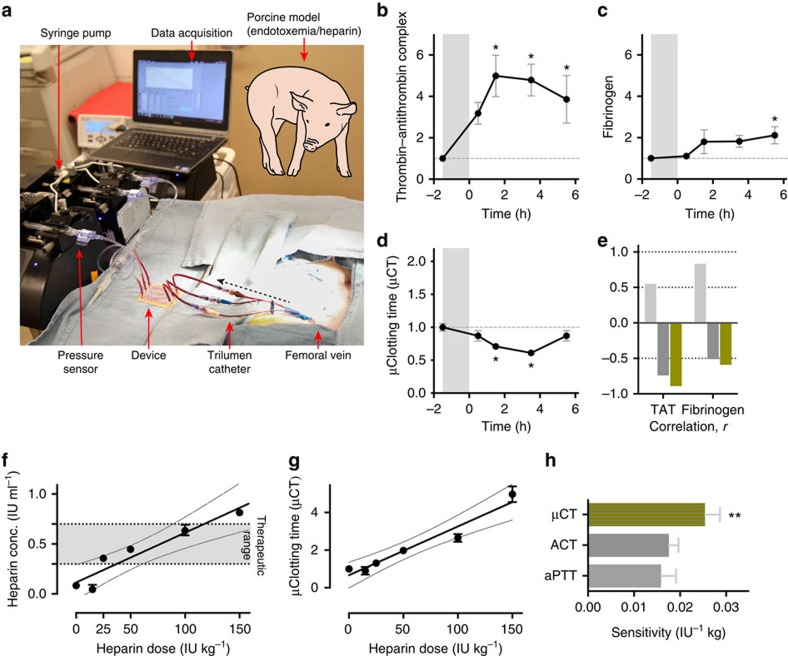
*Ex vivo* monitoring of coagulopathy and anticoagulation therapy in pigs. (**a**) Photograph showing a functioning microfluidic device attached to the femoral vein of a pig via a trilumen catheter. Blood is pulled at a set flow rate (shear gradient=4,375 s^−1^ mm^−1^) so that it passes through the device and the inline pressure sensor, forming clots inside the device; pressure data are automatically acquired on a laptop. (**b**–**d**) endotoxemic shock model. Lipopolysaccharide endotoxin (LPS) was infused into a pig over 1.5 h (grey shaded region, −1.5–0 h), and then thrombin-antithrombin (TAT) complexes in plasma (**b**; baseline=11.95 ng ml^−1^), fibrinogen concentration in plasma (**c**; baseline=143.61 ng ml^−1^), and microfluidic clotting time (μCT) calculated using the microchip (**d**; baseline=20.64 min; *P<0.05 compared with baseline, one-way ANOVA, s.e.m.) were measured every hour thereafter. (**e**) Pearson correlation coefficient (*r*) of aPTT (light grey), ACT (dark grey) and μCT (green) compared with TAT and fibrinogen measurements (*n*=3 pigs, 2 replicates per experiment). (**f**,**g**) Heparin therapy model. Fold change (relative to baseline at no heparin), in heparin concentration (black circles) measured by Factor Xa assay (**f**) and μCT (black circles) measured using the microfluidic haemostasis monitor (**g**) (baseline=4.25 min; full line, line of linear regression; dotted line, 95% confidence interval of the linear fit). (**h**) Graph showing the sensitivity of aPTT (light grey), ACT (dark grey) and μCT (green) assays (**P<0.01, one-way ANOVA, s.e.m.; *n*=3 pigs, 2 replicates per experiment).

Another major unmet need in the haemostasis field is the ability to measure the coagulation status of patients in real time within blood flowing through extracorporeal circuits. Under these conditions, patients commonly require frequent haemostasis monitoring to ensure therapeutic anticoagulation is maintained at effective levels, and to rapidly detect any life-threatening thrombotic or bleeding events that might arise. To explore the potential utility of using this microfluidic device as an *ex vivo* haemostasis monitor, we integrated a three-channel device directly into a vascular access line that was inserted into the femoral vein of a living pig (Fig. 4a) and performed two separate proof-of-concept studies. Animal models of endotoxemia induced by intravenous infusion of lipopolysaccharide (LPS) are widely used to study disruption of haemostasis and intravascular coagulation owing to sepsis^33^ Therefore, we first injected a lethal dose of LPS intravenously to the pig for 90 min and then monitored the coagulation state of the animal's blood for 6 h. Samples were drawn from the venous access line for conventional blood analysis (complete blood count, blood chemistry and coagulation parameters) and the clotting time was also determined by flowing blood from the access line directly through an integrated microfluidic device, which was replaced with a new device every hour over the period of study.

LPS infusion resulted in significant changes in blood cell parameters and haemostasis relative to baseline, including decrease in white blood cell count within 2 h, followed by a rapid increase in white blood cell numbers, as well as an increase in neutrophils, rise in haematocrit, and decrease in platelet counts over 6 h (Supplementary Fig. 3). In addition, activation of the coagulation cascade occurred, as indicated by significant increases in plasma thrombin-antithrombin (TAT) complexes and >2-fold increase in plasma fibrinogen levels over baseline (*P*<0.05; one-way ANOVA) (Fig. 4b,c). Importantly, we also were able to rapidly detect development of this prothrombotic state using the microfluidic device coupled to the vascular access line, as evidenced by a significant decrease in microfluidic clotting time (*P*<0.05; one-way ANOVA; Fig. 4d). Moreover, the trend of our clotting end point over time correlated more closely with these specific plasma biomarkers, when compared with the standard ACT and aPTT coagulation assays, as determined by the Pearson correlation coefficient, *r* (Fig. 4e and Supplementary Fig. 4). In fact, the aPTT resulted in a positive *r*, which indicates that it was highly insensitive to the elevated biomarkers. Moreover, it yielded a trend in clotting time opposite to what would be expected, and thus, it was the worst among the three assays we tested.

In a second study, we injected single doses of different concentrations of unfractionated heparin intravenously into a pig, and determined clotting times sequentially *ex vivo* using a collagen-coated microfluidic device within 10 min after each dose, while also measuring ACT, aPTT clotting times and plasma heparin concentrations in blood samples drawn in parallel. We found that plasma heparin concentration rose linearly in response to increasing heparin doses over the clinical therapeutic range applicable to humans (Fig. 4f)^26^. Heparin dose-dependent increases in aPTT and ACT times were also detected as expected (Supplementary Fig. 5); however, the aPTT test was only sensitive in the lower heparin range and it could not measure effects of heparin when used at doses ∼100 IU kg^−1^ (Supplementary Fig. 5). Importantly, in addition to displaying a linear dose response, the microfluidic clotting times we determined *ex vivo* using the microfluidic device were also significantly more sensitive than those measured using aPTT and ACT tests (sensitivity for all tests was defined as the slope of the linear fit of test value versus heparin dose; *P*<0.01; one-way ANOVA; Fig. 4g,h).

These studies show that the haemostasis assessment microdevice can be successfully integrated into the blood lines of extracorporeal circuits and used *ex vivo* to monitor developing coagulopathy and anticoagulation therapy directly in living patients, which could have great value for children as well as adults. Moreover, this device applied *ex vivo* appears to be more reliable than standard aPTT and ACT laboratory assays, and importantly, it eliminates the need to use anticoagulant collection tubes which further minimizes the chance of pre-analytical errors that complicate existing clinical assays^34^.

## Discussion

The biomimetic haemostasis monitoring device described here provides several potential advantages over other analytical devices and flow chambers that quantitate high-shear thrombosis, although it also has some limitations. First, due to the presence of whole blood flow and stenosed shear gradients in the device, clot formation occurs under near-physiological conditions and includes complex blood rheology that is critical to thrombosis. The device was microfabricated to create a fixed 55% stenosed lesion in each channel that cannot change during the course of the study, as might occur *in vivo*; however, the user can still apply shear rate gradients to mimic patient-specific medical conditions. For example, analysis of blood from atherosclerotic patients who have a higher degree of *in vivo* stenosis documented clinically (for example, by imaging) might be carried out using higher shear gradients that better correspond to the severity of the condition.

Another major advantage of this screening device is that real-time evolution of blood clots can be recorded and quantified easily using just a blood pump, a disposable pressure sensor and a display monitor (imaging equipment is optional), all of which are also part of most extracorporeal circuits and hence, currently used widely in medical settings^35^,^36^. In fact, the blood can be pumped in two operating modes—constant flow rate—where exponential growth of pressure is recorded, or alternatively, constant pressure where sigmoidal decay in flow is measured. Blood could be drawn from the high-pressure arterial line directly into the device, thus further eliminating the need of a blood pump. The feasibility of this approach has been demonstrated by using this device with a constant pressure pump to measure sigmoidal changes in flow across a microchannel as a way to evaluate an antithrombotic surface coating that could potentially be used in extracorporeal circuits^37^.

Using this device, we also were able to identify a microfluidic clotting time that serves as an automated, robust and physiologically relevant analytical end point of this assay by applying a highly predictive phenomenological mathematical model to the biomimetic occlusion measurements. This derived clotting time has a high dynamic range to monitor heparin therapy *in vitro*, and it shows an exponential decay to increasing shear gradients mapping the sensitivity to platelet thrombi formation under flow, making this device well suited to explore whether novel anti-coagulants and anti-platelet drug candidates produce different behaviours in clotting dynamics. Importantly, using this model-based approach, we required <3-fold rise in pressure drop to reliably measure haemostasis, which minimizes the time and blood volumes that are required to carry out these studies.

Another unique feature of our device is the incorporation of multiple parallel stenosed channels. We chose this design to better mimic clotting within a natural blood vessel network than using one very long channel, which would more closely resemble a single occluded vessel. This design is analogous to clotting within multiple vessels of a vascular network (that is, instead of a single vessel) that occurs in living patients with coagulopathies *in vivo* where clots may form, deform, translocate and detach locally, but the pressure still increases in the vascular bed due to the systemic response to thrombosis. Use of multiple parallel channels, combined with our mathematical model that acts as a digital filter, also allows the device to overcome the granularity of responses that can occur in single channels (for example, due to occasional passing of debris, air bubbles or other local perturbations), and provide a more robust global measurement of system-level coagulation behaviour and platelet function. In addition, this design allowed us to maximize the number of stenosed regions and the total surface contact area available to promote blood clot formation. We were also able to fit multiple replicas of the entire device on a microscope slide in this initial characterization study so that we could visualize thrombus formation and validate the results obtained with our pressure measurements. Finally, the 12-channel design generated a dynamic range of pressures that were easily measured using our commercially available pressure sensor. However, we have not optimized the parallel network design, and with different design constraints it is possible that other channel designs, or even use of single channels, could be feasible. Thus, further design optimization will need to be carried out if this technology is advanced towards clinical applications.

This device also had limitations, however. For example, the time to assess clotting is not instantaneous and it does not permit continuous monitoring of clotting in flow loops as each device would need to be discarded after every sequential measurement. But most existing haemostasis assays are also not instantaneous, and clinical decisions are often made over the time span of many hours. Thus, the 10–20 min that are required for completion of this assay by the bedside using disposable analytical components that can be snapped in and out over time should still be valuable for clinical analysis.

One of the major features of our device is that it also can effectively monitor platelet function in whole blood, which can be applied to analyse bleeding risk in patients who are not easily diagnosed. Interestingly, being a global and quantitative haemostasis test, this device offers a potential way to analyse more complex coagulopathies, such as sepsis and sickle cell anaemia, where other cells (for example, bacteria and sickled erythrocytes, respectively) also contribute to alterations in haemostasis. Moreover, because this device can be functionally integrated with extracorporeal circuits and indwelling medical devices as we showed here, it could provide a relatively simple and more reliable way to monitor coagulopathies and effects of antithrombotic therapy over time using native blood in both critical care or home care settings.

Finally, we have also gained some interesting new insights into the biophysical process of thrombus formation through use of this device. For example, while increased coagulation has been observed in regions of living vessels that experience high-shear rates^25^ and shear rate gradients^14^ in the past, it was not possible to determine which of these parameters is the most critical trigger for coagulation. In contrast, our ability to independently vary shear rate gradients using our microfluidic device design allowed us to show that the shear gradient in the post-stenosed (decelerating flow) region is the more critical activator of coagulation, and that the overall microfluidic clotting time shortens exponentially as the applied shear rate gradient increases. The cause of this response is unknown, but it is likely due to the activation of clotting factors, platelets and/or immune cells via fluid dynamics-dependent mechanical signalling pathways that remain to be defined. In any case, these findings suggest that this microfluidic device may be useful for mechanistic studies, in addition to serving as a potential clinical diagnostic device.

## Methods

### Microdevice fabrication

The devices were designed to fit on a standard (75 × 50 mm) microscope slide to simplify microscopic analysis using the AutoCAD software and we used SU8 2075 (MicroChem. Corp.) master templates fabricated on Si (100) wafers (University Wafer Corp.) using photolithography. The devices were fabricated using soft lithography of PDMS^38^. Slygard 184 PDMS prepolymer (Dow Corning) was cast on the silanized master which had the positive relief of the channel features formed by the SU8 photoresist. The PDMS was then cured at 60 °C in a convection oven for 3–4 h. The cured PDMS was peeled off the master and bonded to a 500-μm-thick PDMS-coated glass slide after treating both with oxygen plasma (100 W, 30 s) and punched with a 4 mm hole at the inlets and outlets (Harris Uni-Core, VWR).

### Principle of coagulation monitoring

Fresh human blood stored in a 5 ml syringe (BD) or a fluid reservoir is pushed or pulled via syringe pump (PHD Ultra CP, Harvard Apparatus) through an inline, disposable pressure sensor (PendoTECH) followed by the PDMS device, respectively. The channel occlusion is measured by recording the rise in pressure over time (5 kPa or above) using data acquisition and analysis software (Winwedge Pro, TALtech). Polyvinyl chloride tubing connected to female luer lock and male luer slip fittings on either ends, respectively, (Qosina Corp, Edgewood, NY) is directly bonded (luer slip end) to the inlet and outlet ports of the device, respectively. The latter end of the inlet side tubing is connected to the pressure sensor and the outlet side of the tubing is dipped in 3.2% sodium citrate (Fig. 1a,b). To significantly reduce clot formation inside the syringe, sensor and tubing, we treated the blood-contacting surfaces with a bioinspired surface coating described elsewhere^37^. When the blood was filled in a syringe and pushed, the syringe was manually agitated every 2–3 min to prevent sedimentation of erythrocytes in the blood. In studies where heparin blood was used, different shear rates and gradients were applied to determine the validity of the clotting time analysis and analyse the response to shear changes. Recalcified citrated blood from patient samples were used for platelet function analysis. All studies were performed at a shear rate gradient of 4,375 s^−1^ mm^−1^ to cause rapid clotting within a few minutes and to limit the patient blood volume required to <0.5 ml. Thrombus formation was observed using time-lapse imaging (× 10, numerical aperature 0.3; Zeiss Axio 3 Observer) of fluorescently labelled fibrinogen (150 mg ml^−1^, Alexa Fluor 488, Invitrogen) and platelets. Human CD41-PE antibody (10 μl ml^−1^, Invitrogen) was added directly to the blood and incubated at room temperature for 10 min for platelet imaging. Standard wide-field fluorescence micrographs of fibrin and platelet staining were recorded to demonstrate total thrombus volume in the different regions of the device; multiple fluorescent microscopic images recorded from neighbouring regions using automatic scanning were also stitched together to form large region panoramas. For data recording and analysis, the initial time (*t*=0) was the time when the blood entered the device. The heparin concentration and flow rate were varied independently in these experiments. The wall shear stress/rate corresponding to the imposed flow rate at the uniform width region was determined from analytical formulae derived for rectangular microchannels^24^ and shear gradients at the pre-stenosed and post-stenosed regions were predicted from computational analysis.

### Computational analysis of shear rate gradients

Finite element-based software, COMSOL Multiphysics v5 (COMSOL Inc, Burlington, MA) was used to simulate blood flow inside the microdevice. The AutoCAD line drawing of the entire device in.dxe format was exported to COMSOL. Blood was assumed to be a two-dimensional incompressible fluid, homogenous and non-Newtonian. A high-density finite element mesh was constructed for precise calculations. The Navier–Stokes equation of fluid dynamics was solved by assigning a finite velocity as the inlet boundary condition, and constant atmospheric pressure (*P*=0) at the outlet boundary. To describe the stress-strain relationship and blood viscosity, a generalized power-law constitutive equation for non-Newtonian fluids was applied and parameters were chosen based on published values^39^,^40^. COMSOL was able to calculate and export the values of wall shear rates along the selected boundary edges during post processing. Shear gradients were calculated by computing the slope of the shear rate near the pre-stenosed and post-stenosed regions.

### Mathematical formulation of clotting dynamics

We developed a mathematical formulation of clot formation dynamics based on the sigmoidal dynamics of thrombus formation observed on blood perfusion inside the device (Fig. 2b). Based on the previous studies showing that the size of a growing thrombus measured *in vitro* correlates linearly with measured light intensity^41^,^42^, we can approximate that 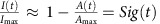, where *A*(t) is the cross-sectional area available for blood flow through the occluding channel at a given time, *A*_max_ is the initial cross-sectional area of the microchannel and Sig(*t*) is a sigmoidal function. Further, since the hydraulic resistance (*R*_h_) of the occluding microchannel approximately scales as 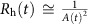 (ref. 43) and our device has parallel sections and every section will not occlude equally at the same time, the hydraulic resistance of the whole device would scale as 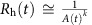. This leads to the relationship, 
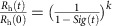
. Further, based on the Hagen–Poiseuille law (*Q*=Δ*P*/*R*_h_) of laminar flow, where *Q* is flow rate and *ΔP* is pressure drop across the device, we arrive at the following general relation to predict the rise in pressure drop of an occluding device when constant flow is applied:


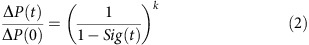


and


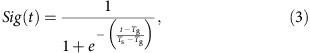


where *T*_g_ and *T*_s_ are the characteristic parameters of the two-parameter sigmoid function. Equations (2) and (3) lead to an exponential growth curve (reciprocal of sigmoid) for pressure, of the form


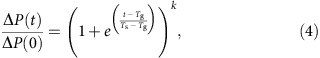


where *T*_g_ and *T*_s_ represent time for the pressure to double and (1+e) times its initial value, respectively. The measured rise in pressure drop is not sensitive to parameter *k* (Supplementary Fig. 6), and therefore, for simplicity and to reduce one dimension from the equation (4), we assume *k* to be unity, which leads to the approximation that the rise in pressure drop of an occluding microchannel follows a two-parameter exponential growth curve described in equation (1).

### Collagen coating of microdevices

To activate the surface, the device was first treated with oxygen plasma for 45 s at 100 W followed by infusion of 2% solution of (3-aminopropyl) trimethoxysilane (Sigma). The 2% (3-aminopropyl) trimethoxysilane is prepared in 200 proof anhydrous ethanol (Sigma). Then, the device was washed with 140 proof ethanol followed 200 proof ethanol (Koptec) and then left to dry at 60 °C for a few hours in a convection oven. Rat tail type I collagen (Corning) was diluted in PBS and amine coupling reagent, N-(3-Dimethylaminopropyl)-N′-ethylcarbodiimide hydrochloride (20 mg ml^−1^; Sigma) in a 1:1 ratio to reach a final concentration of 100 μg ml^−1^ and infused into the device. The devices were incubated at 37 °C for 3–4 h and then washed with 1% bovine serum albumin solution followed by saline or PBS, before blood perfusion.

### Blood samples

Blood samples were collected after informed, written consent as per ethical guidelines of Institutional Review Board of Partners Healthcare, Harvard Medical School and Beth Israel Deaconess Medical Center. Healthy donors: blood was collected from non-smoking healthy volunteers in a standard 6 ml no-additive blood vacutainer (BD) and 1000 U ml^−1^ unfractionated heparin was immediately added to a required concentration. Coagulation experiments were initiated within ∼15 min after the blood draw. For experiments where heparin concentration was <0.25 IU ml^−1^, blood was first drawn in 3.2% 5 ml sodium citrate vacutainers (BD) or purchased (Research Blood Components, Cambridge, MA). Patients: subjects who were taking aspirin and plavix (clopidogrel) were selected from among patients seen in the cardiac catheterization laboratory at Beth Israel Deaconess Medical Center, Boston, MA. Whole blood from patients with HPS was drawn using standard phlebotomy techniques and stored in 4.5 ml citrated (3.2% sodium citrate) tubes at room temperature. Citrated blood was used within 5 h of blood draw. The coagulation activity of these samples was restored by adding 100 μl ml^−1^ of 100 mM calcium chloride/75 mM magnesium chloride solution.

### Anti-platelet drug

Abciximab (Reopro) was purchased from Boston Children's Hospital and diluted in saline to a desired concentration before adding to whole blood.

### Animal studies

All animal studies were approved by the Institutional Animal Care and Use Committee at Boston Children's Hospital and carried out in an Association for Assessment and Accreditation of Laboratory Animal Care accredited, United States Department of Agriculture (USDA) registered facility. Female Yorkshire pigs (25–35 kg; 3–4 months old) were acclimatized for at least 72 h before experimentation. The animal was anaesthetised using standard procedures after which the bilateral femoral veins were percutaneously cannulated with 5–8 French triple lumen catheters (Arrow International, Reading, PA). The catheters were rinsed using multiple saline flushes before use, and no catheter line was used more than twice. A femoral artery was similarly catheterized for direct measurement of mean arterial blood pressure. The microfluidic devices were connected to the catheters via luer locks. The blood was pulled at a rate corresponding to the shear rate gradient of 4,375 s^−1^ μm^−1^. Endotoxemia: baseline measurement of coagulation parameters were performed followed by infusion of LPS endotoxin (*E. Coli* O26:B6 strain, Sigma) at a rate of 0.1–0.2 μg kg^−1^ min^−1^ or 60–90 min reaching an approximate total dose of 10 μg kg^−1^ (ref. 44). Fluid therapy was maintained to support the blood pressure >50 mm Hg. At the end of LPS infusion, devices were operated to measure microfluidic clotting time. Five to fifteen minutes later, in parallel to the devices, blood was drawn from one of the catheter ports for aPTT/ACT/complete blood count (CBC)/Lab analysis. The experiment was repeated (a new device was attached and old one discarded) every hour for up to 6 h post-LPS infusion, allowing us to study and validate progression of coagulopathy in this animal model. Heparin therapy: the microfluidic device was connected to the catheter and then, a bolus dose of unfractionated heparin (0–150 IU kg^−1^) was given to the animal intravenously. The device was operational within 5–10 min of the injection and ran until the analytical endpoint was reached for each dose (pressure across the device rose five times the initial value or flow was reduced to nil inside the device or after the passage of 30 min). In parallel, blood samples were obtained for ACT/aPTT/CBC/lab analysis from one port of the multi-lumen catheter at 15 min after the heparin injection. After each injection of heparin and the clotting analysis with devices, the animal was brought back to baseline haemostasis confirmed by monitoring aPTT/ACT values every hour. Once haemostasis was restored, another heparin dose was delivered to the same animal and a new experiment was conducted with a new device attached. After recursively testing the clotting times at various heparin doses in this way, at the end of the study, the animal was euthanized under anaesthesia with injection of Fatal Plus.

### Plasma biomarkers

Factor Xa: heparin was determined in blood platelet poor plasma using a Factor Xa chromogenic kit (Chromogenix Antithrombin, Diapharma Group, West Chester, Ohio)^45^. TAT complex: TAT using a sandwich enzyme-linked immunosorbent assay (ELISA) assay were measured in plasma using manufacturer's instructions (Enzygnost TAT Micro Kit, Siemens Healthcare). Fibrinogen was measured with ELISA assay using manufacturer's instructions (Fibrinogen porcine ELISA kit, ALPCO Immunoassays, Salem, NH).

### Clotting time laboratory assays

CBC was performed in duplicate in 1 ml EDTA blood vacutainers (VetScan HM5, Abaxis, Union City, CA). aPTT was performed in duplicate cartridges using citrated blood (VetScan VSpro, Abaxis, Union City, CA). ACT was performed in duplicate using ACT tubes provided by the manufacturer (Hemochron, Accriva Diagnostics). EDTA and citrate vacutainers were manufactured by Becton Dickinson.

### Scanning electron microscopy

The PDMS to PDMS bonded devices were peeled off manually after thrombus formation. The post-stenosed region of the device was cut in an ∼1 × 1 cm size and fixed with 2.5 % glutaraldehyde in 0.1 M sodium cacodylate buffer (Electron Microscopy Sciences, Hatfield PA) for 1 h, 1 % osmium tetroxide in 0.1 M sodium cacodylate (Electron Microscopy Sciences, Hatfield PA) for 1 h, dehydrated in ascending grades of ethanol, and chemically dried with hexamethydisilazane (Electron Microscopy Sciences, Hatfield PA). Samples were sputter coated with 5 nm gold and imaged on a Zeiss Supra55VP microscope.

### Statistical analysis

*In vitro* assay sample size was predetermined with three separate healthy donors, nine subjects taking anti-platelet medication and two patients with a rare HPS bleeding deficiency to account for biological variability. For *ex vivo* studies, a sample size of three pigs was used to account for biological variability. Blood samples were excluded only if they were >6 h old before the study was conducted, and microfluidic devices were excluded due to fabrication abnormalities, such as leakage. The goodness of fit was quantified by the sum of squares, from the best-fit curve determined by nonlinear regression (*R*^2^) or linear regression (*r*^2^). Data analysis, curve fitting and goodness-of-fit analysis were performed using Graphpad Prism V6.

## Additional information

**How to cite this article:** Jain, A. *et al*. A Shear Gradient-Activated Microfluidic Device for Automated Monitoring of Whole Blood Haemostasis and Platelet Function. *Nat. Commun*. 7:10176 doi: 10.1038/ncomms10176 (2016).

## Supplementary Material

Supplementary Supplementary Figures 1-6 and Supplementary Table 1

Supplementary Movie 1 Time-lapse imaging of fluorescent fibrinogen in whole blood containing 0.75 IU ml-1 heparin anticoagulant and an applied flow rate corresponding to shear rate gradient of 4,375 sec-1mm-1. Each figure is a scan of 4 X 1 tiles (10X objective, 0.3 N.A.) stitched together. Each frame is taken at an interval of 1 minute. For presentation, the movie speed is set to 7 frames per second.

## Figures and Tables

**Table 1:** Clotting time analysis of patients with Hermansky–Pudlak Syndrome (HPS).

Shear gradient (s^−1^ mm^−1^)	4,375	8,750	4,375	4,375	4,375	4,375
ADP (μM)	0	0	1	5	7.5	10
μClotting Time (min; Patient 1)	N	N	N	N	9.45	6.23
μClotting Time (min; Patient 2)	N	N	N	N	7.67	4.56

Mean of three replicates per experiment. N: blood did not clot in the device.
