# Supplementary material for: A shear gradient-activated microfluidic device for automated monitoring of whole blood haemostasis and platelet function
Source: Nat Commun. 2016 Jan 6;7:10176. doi: 10.1038/ncomms10176 (PMC4729824; doi:10.1038/ncomms10176)
Supplement: Supplementary — Figures 1-6 and Supplementary Table 1 [file ncomms10176-s1.pdf]

SUPPLEMENTARY FIGURES

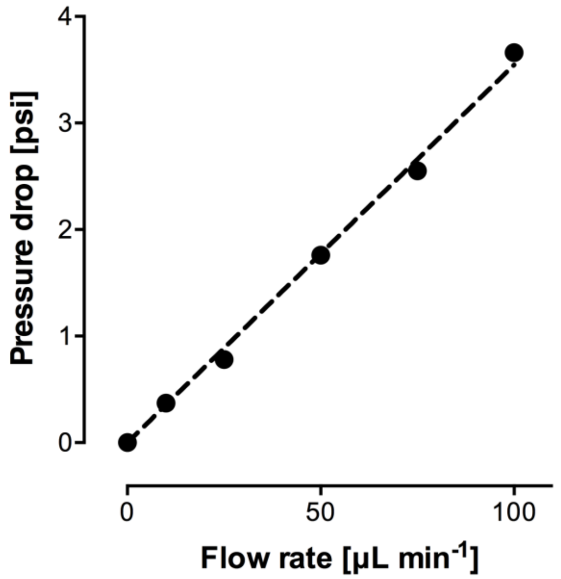

Supplementary Figure 1. **Analysis of dynamic range of measurement**. Pressure drop measured via inline pressure sensor vs applied flow rates when whole blood drawn in sodium citrate is perfused through the device containing 12-parallel stenosed microchannels (circle). The dotted lines indicate linear curve fit to measured data. For a 12-lane device, the pressure drop is in the range 0.4 – 3.5 psi and if the anticoagulation is removed and blood is allowed to clot, the pressure drop inside the device could reach 1.5 – 12 psi. This dynamic range is suitable to measure hemostasis using the inline pressure sensor that operates within 0-30 psi pressure range (2 individual experiments were performed).

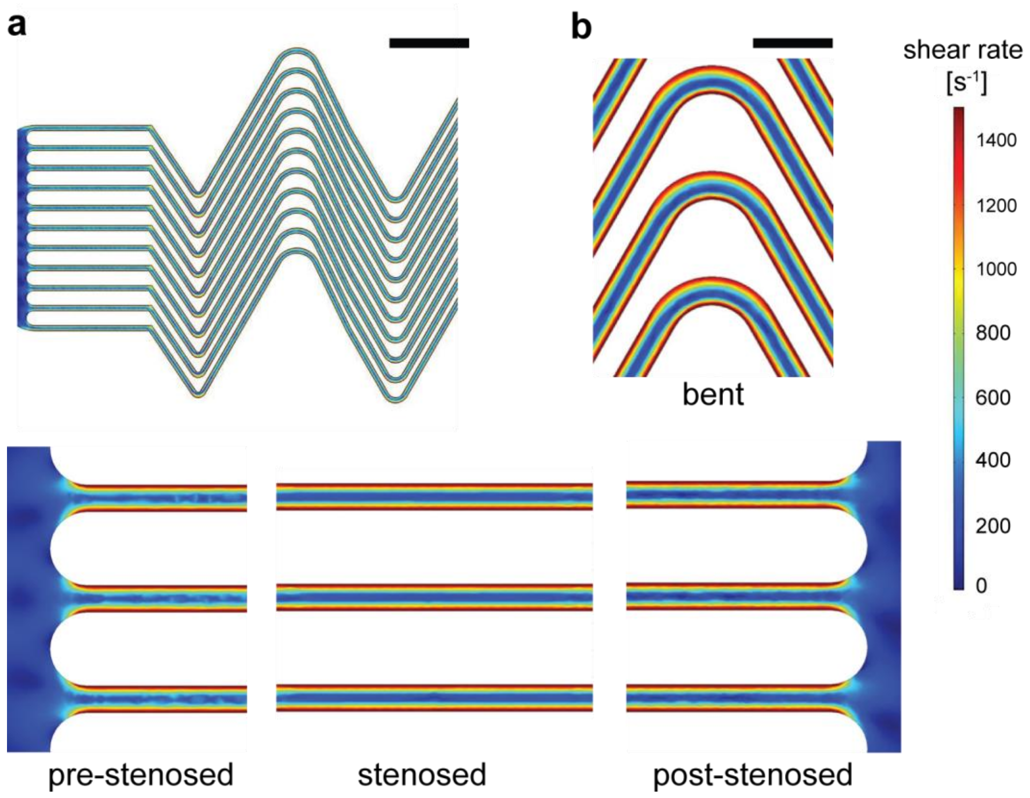

Supplementary Figure 2. **Shear rate distribution in the microdevice.** A representative heat map showing shear rate (s^{-1}) distribution in **a**) a large section of the device (bar, 1 mm), and **b)** bent, pre-stenosed, stenosed and post-stenosed sections of the device, at an inlet velocity of u = 0.2 m s^{-1} (bar, 400 μm).

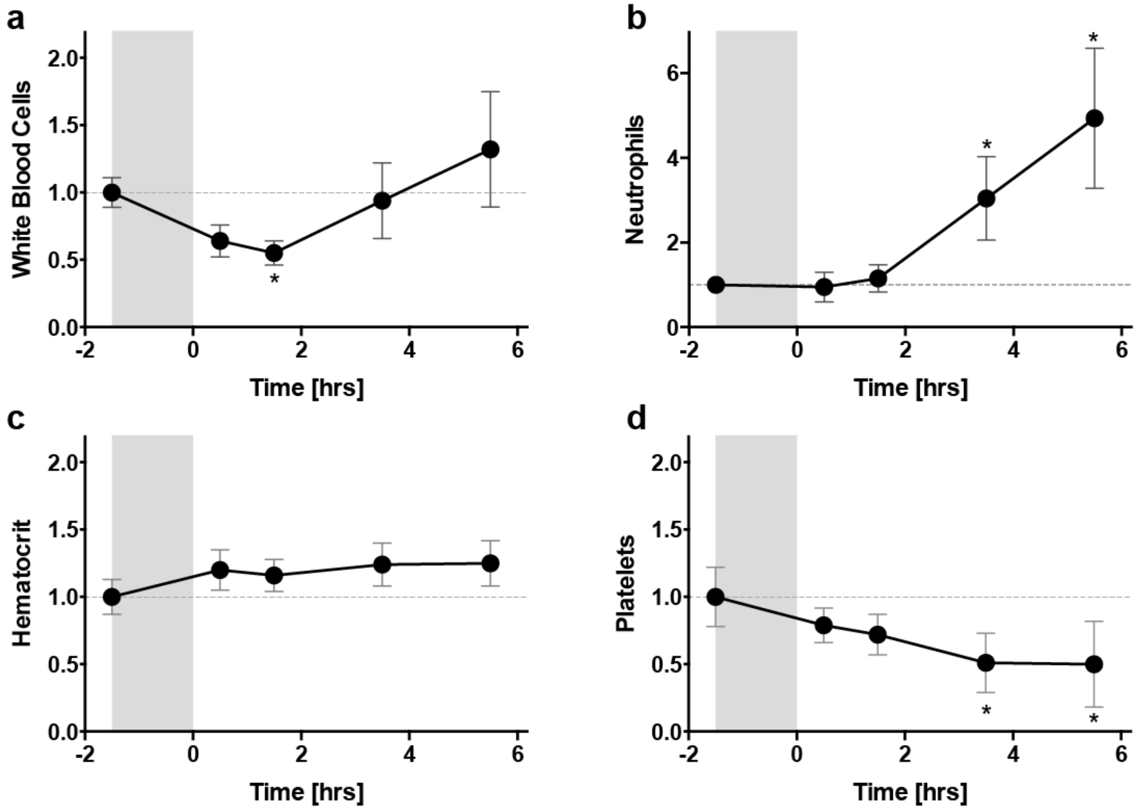

Supplementary Figure 3. Blood cell count in coagulopathy. Fold change (relative to baseline (------) at -1.5 hours) in **a)** white blood cell count (mean baseline = 10.41 X 10^9/L), **b)** neutrophil count (mean baseline=2.47 X 10^9/L), **c)** hematocrit (baseline = 30.13%) and **d)** platelet count (baseline=281.5 X 10^9/L), during 6 hours of monitoring in porcine model of endotoxemia (n = 3 pigs, measured in duplicate and averaged at each time point). *$P<0.05$ vs baseline; one-way ANOVA, s.e.m.

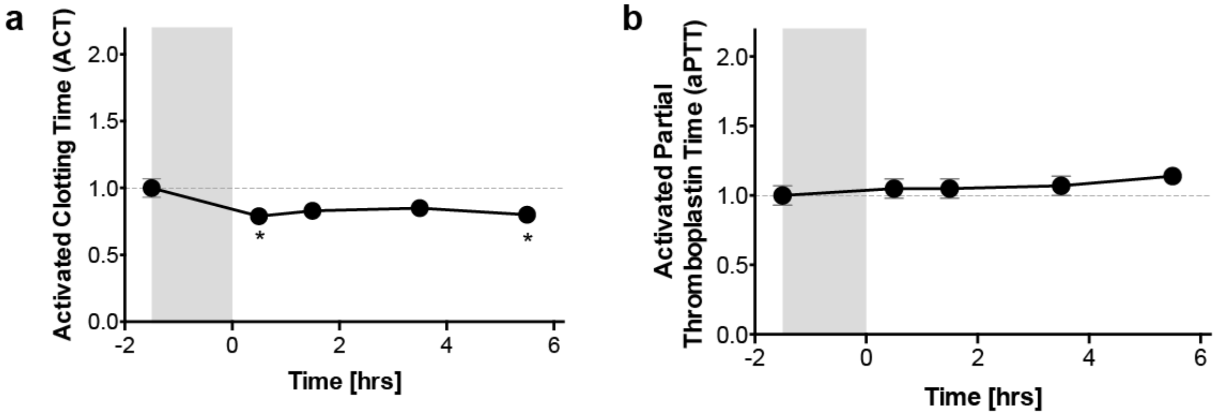

Supplementary Figure 4. Standard coagulation assays in coagulopathy. Fold change (relative to baseline (- - -) at -1.5 hours) **a)** ACT (mean baseline = 126.50 s), and **b)** aPTT (mean baseline = 78.45 s), during 6 hours of monitoring in porcine model of endotoxemia (n = 3 pigs, measured in duplicate and averaged at each time point)**.** *P<0.05 vs baseline, one-way ANOVA, s.e.m.

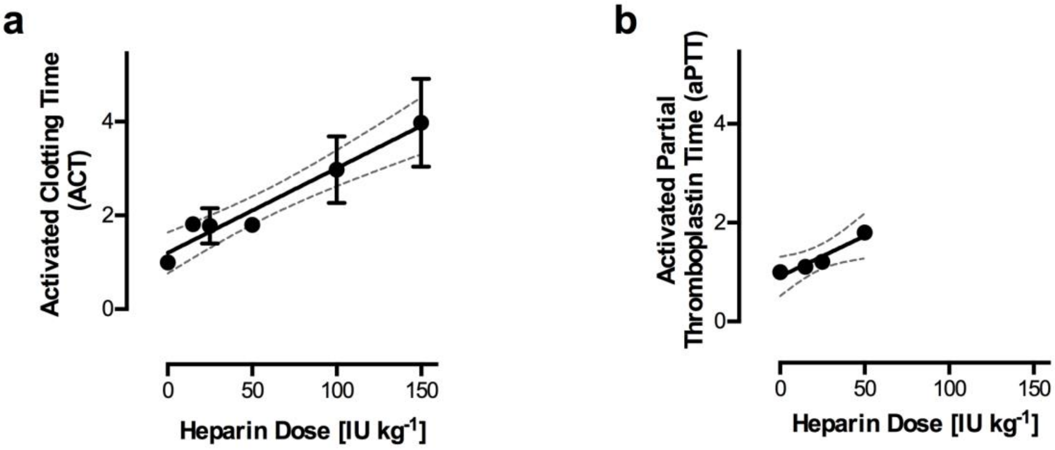

Supplementary Figure 5. Standard coagulation assays in heparin therapy. Fold change (relative to baseline at no heparin) **a)** ACT (mean baseline = 119.63 s, and **b)** aPTT (mean baseline = 81.35 s), at various therapeutic heparin doses injected to the pig (n = 3 pigs, measured in duplicate and averaged for each heparin dose). Line of linear regression (—), region of 95% confidence interval (- - -). error bar, s.e.m.

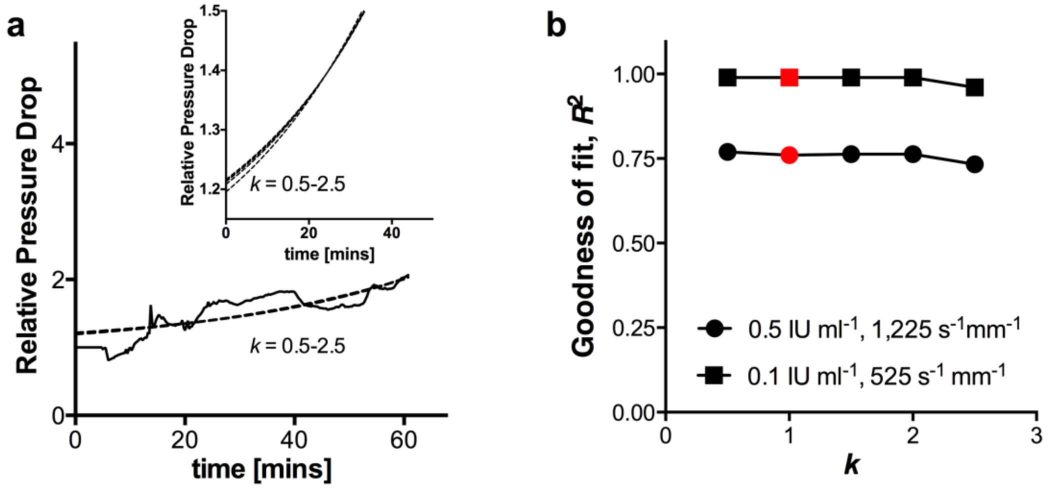

Supplementary Figure 6. Sensitivity analysis of parameter, *k*, in the range [0.5-2.5], to the goodness of fit, R^2. a) Regression curves at variable *k* values shown for a representative condition of rise in pressure drop when blood was perfused through the device (0.5 IU ml^{-1}, 525 s^{-1} mm^{-1}). Solid line is experimental data and dotted lines are the regression curve fit. **b**) Goodness of fit, R^2, versus *k* showing that curve-fits for two datasets, (0.5 IU ml^{-1}, 525 s^{-1} mm^{-1}) and (0.1 IU ml^{-1}, 1,225 s^{-1} mm^{-1}), are not sensitive to the parameter *k*. Therefore, *k* = 1 (red) is a good approximation to determine microfluidic clotting time.

Supplementary Table 1. Goodness of Fit parameter, R^2, of respective curve fits at various shear rate gradients and heparin concentrations. The mean value from three experiments rounded to two decimal places is reported. NA: Not Applicable

Heparin [IU ml^{-1}]	Shear Rate Gradients [$s^{-1}mm^{-1}$]					
	262.5	525	1,225	2,625	4,375	8,750
0	NA	NA	0.99	NA	0.99	NA
0.1	NA	NA	0.99	NA	0.97	NA
0.25	NA	0.91	0.92	0.98	0.86	0.9
0.5	NA	0.77	0.76	0.88	0.83	0.82
1	NA	NA	0.84	NA	0.89	NA
